# Scoliosis in dysplastic spondylolisthesis: a clinical survey of 50 young patients

**DOI:** 10.1186/s12891-022-05297-7

**Published:** 2022-04-08

**Authors:** Xinhu Guo, Zhaoqing Guo, Weishi Li, Zhongqiang Chen, Yan Zeng, Woquan Zhong, Zihe Li

**Affiliations:** Department of Orthopaedics, Peking University Third Hospital, Beijing Key Laboratory of Spinal Disease Research, Beijing, 100191 China

**Keywords:** Dysplastic spondylolisthesis, Developmental spondylolisthesis, Adolescent idiopathic scoliosis, Spasm scoliosis, Olisthetic scoliosis

## Abstract

**Background:**

Dysplastic spondylolisthesis is a rare spinal deformity that occurs mainly in young patients. Although its sagittal parameters had been well stated, coronal abnormalities in these patients were poorly studied. The purposes of this study were: (1) to investigate the prevalence of scoliosis in dysplastic spondylolisthesis;(2) to assess scoliosis resolution or persistence after surgery; and (3) to propose a modified classification of scoliosis associated with dysplastic spondylolisthesis.

**Methods:**

Fifty patients (average age 14.9 ± 5.6 years) diagnosed with dysplastic spondylolisthesis who underwent surgical treatment were followed up and their data were analyzed. Standing posteroanterior and lateral full spine radiographs were used to measure the coronal and sagittal parameters. Patients with scoliosis, which was defined as a coronal Cobb angle greater than 10°, were divided into three groups according to their curve characteristics: “independent” scoliosis (IS) group, spasm scoliosis (SS) group, and olisthetic scoliosis (OS) group. SS and OS were spondylolisthesis-induced scoliosis. The radiographic parameters and patient-reported outcomes were collected before and after surgery and compared between groups.

**Results:**

The average slip percentage was 62.8% ± 23.1% and the average follow-up time was 51.5 ± 36.4 months (range 3–168 months). Twenty-eight of the 50 (56%) dysplastic spondylolisthesis patients showed scoliosis, of which 8 were IS (24.7° ± 15.2°), 11 were SS (13.9° ± 3.0°), and 9 were OS (12.9° ± 1.9°). By the last follow-up, no scoliosis resolution was observed in the IS group whereas all SS patients were relieved. Of the nine patients with OS, four (44.4%) had scoliosis resolution after surgery.

**Conclusion:**

Distinguishing different types of scoliosis in dysplastic spondylolisthesis patients may help surgeons to plan treatment and understand prognosis. For patients with significant scoliosis, whether “independent” or spondylolisthesis-induced, treatment of spondylolisthesis should be performed first and scoliosis should be observed for a period of time and treated according to the corresponding principles.

## Background

Dysplastic spondylolisthesis is a rare spinal deformity that occurs mainly in young patients and typically involves L5-S1. It is characterized by major dysplasia or malformation of the arch–facet configuration and progression is more likely than in isthmic spondylolisthesis [1]. Furthermore, it often leads to lumbosacral kyphosis and abnormal spino-pelvis alignment [1, 2]. Although previous studies have focused on the characteristics of the sagittal parameters of dysplastic spondylolisthesis, little research has paid attention to coronal abnormalities [2–6].

**Fig. 1:**
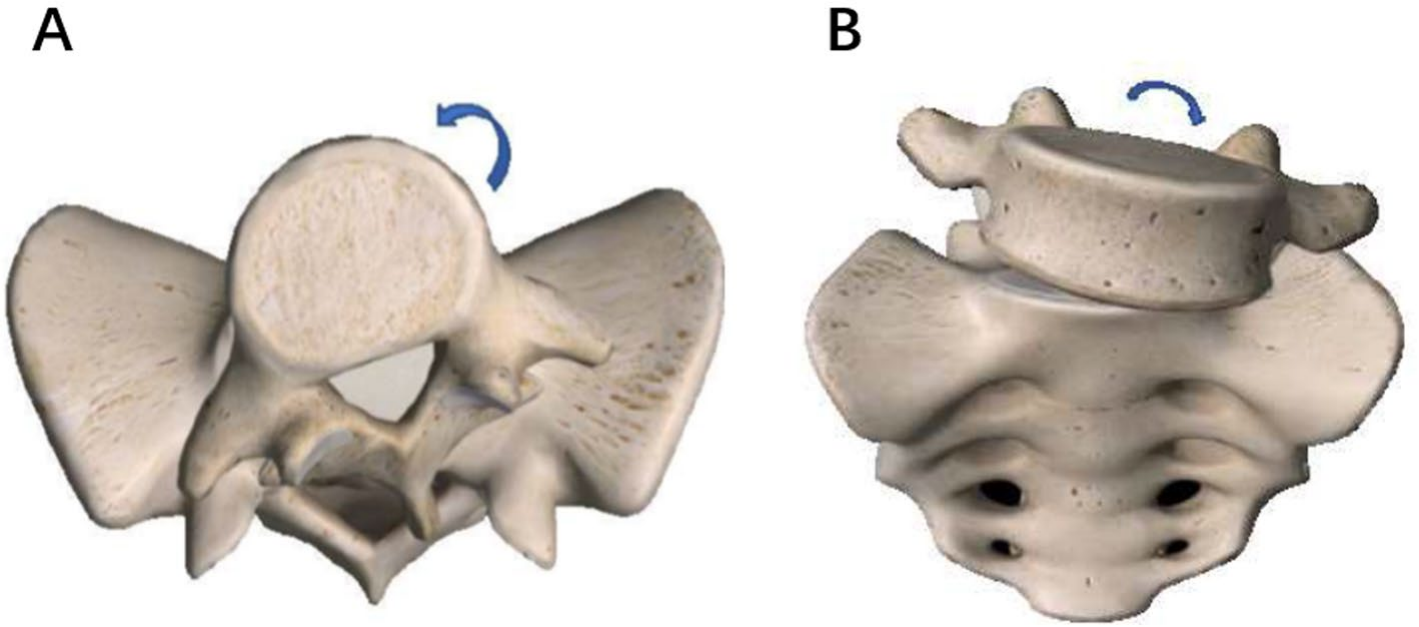
The mechanism of the development of olisthetic scoliosis, showing the rotation (**A**) of L5 on the axial plane and the tilt (**B**) of L5 on the coronal plane during slipping

The estimated prevalence of scoliosis in the adolescent population is 0.47–5.2% [7], whereas it is 18–48% in young patients with lumbar spondylolisthesis [8–11], suggesting that spondylolisthesis may induce scoliosis by some mechanism. Two main types of spondylolisthesis-induced scoliosis have been defined in the literature: spasm scoliosis (SS) and olisthetic scoliosis (OS) [8–13]. SS is thought to be caused by muscle spasm and similar to scoliosis associated with other painful spine pathologies, like disk herniation, with typical listing of the spine to the side [8, 12, 13]. OS, also termed torsion scoliosis, is associated with asymmetric slippage and sink of the olisthetic vertebra, with more rotation in the olisthetic vertebra [11] (Fig. 1). From literature, patients with dysplastic spondylolisthesis appear to have a higher rate of scoliosis than patients with isthmic spondylolisthesis [8, 10], suggesting that, in addition to the sagittal plane abnormality, dysplastic spondylolisthesis patients are prone to coronal plane abnormality. The present study reviewed a consecutive series of L5-S1 dysplastic spondylolisthesis patients treated with complete or partial reduction with short-segment fixation and fusion. The purposes of this study were: (1) to investigate the prevalence of scoliosis in dysplastic spondylolisthesis; (2) to assess scoliosis resolution or persistence after surgery; and (3) to propose a modified classification of scoliosis associated with dysplastic spondylolisthesis.

## Methods

We performed a retrospective study of patients diagnosed with dysplastic spondylolisthesis between March 2007 and November 2020 at the orthopaedic department of a tertiary hospital. The inclusion criteria were: (1) age ≤ 30 years when admitted to hospital; (2) a diagnosis of L5-S1 dysplastic spondylolisthesis according to the Wiltse classification of spondylolisthesis: Dysplasia or malformation of the arch-facet configuration at L5-S1 that lead to instability and anterior translation [1]; All the patients were diagnosed of high-dysplastic spondylolisthesis in Marchetti and Batolozzi classification system according to Mac-Thiong’s criteria [14] and (3) availability of preoperative radiographic and quality of life (QOL) data with a minimum follow-up of three months after surgery with radiographic and QOL data. Patients who were lost to follow up and with previous spinal surgery, tumor, or infection were excluded.

A total of 50 young patients (six males and 44 females) with an average age of 14.9 ± 5.6 years (range 7–30 years) were included in this study. According to the Meyerding grading scale, the cohort included 2 grade I, 13 grade II, 19 grade III, 13 grade IV, and 3 grade V spondylolisthesis cases and the average slip percentage was 62.8 ± 23.1%. The patients had the following surgical indications:(1) 44 cases had low back pain and/or lower extremity pain that failed to respond to conservative treatment; and (2) six cases had cauda equina syndrome. Posterior decompression, partial or complete reduction of L5, L5-S1 intervertebral fusion (including disc resection, sacral dome resection and autogenous bone grafting) and instrumentation of L4-S1 or L5-S1 were performed in 48 patients. L5 resection with L4-S1 or L3-S1 instrumentation and fusion was performed in two patients using the combined anterior and posterior approach. All patients were followed up for more than 1 year, except one patient who was only followed up for 3 months. The average follow-up time was 51.5 ± 36.4 months (range 3–168 months).

Standing posteroanterior and lateral full spine radiographs were used to measure the coronal and sagittal parameters. The coronal parameters were as follows: (1) coronal Cobb angle of the main curve, the angle between the superior endplate of the upper end vertebrae and the inferior endplate of the lower end vertebrae; (2) CSVL-C7PL, the horizontal distance between the C7 plumb line (C7PL) and the central sacral vertical line (CSVL); and (3) curve span, the number of vertebrae included in the main curve. Scoliosis was defined as a Cobb angle > 10°. A CSVL-C7PL > 20 mm was defined as coronal imbalance. Scoliosis resolution was considered if the Cobb angle was < 10° at last follow-up. The following sagittal spinopelvic parameters were analyzed: (1) pelvic incidence (PI), defined as the angle between a line joining the center of the upper endplate of S1 to the axis of the femoral heads and a line perpendicular to the upper endplate of S1; (2) pelvic tilt (PT), the angle between the vertical line and a line drawn from the center of the upper endplate of S1 to the axis of the femoral heads; (3) sacral slope, the angle between the upper endplate and the horizontal line; (4) lumbar lordosis (LL), defined as the angle between the upper endplate of L1 and the upper endplate of L5 as LL, because the dome-shaped S1 upper endplate made it difficult and inaccurate to measure L1-S1 lumbar lordosis; (5) sagittal vertical axis (SVA), the distance between the plumb lines dropped from the center of the C7 vertebral body and the posterior-superior aspect of the S1 vertebral body; and (6) Dubousset’s lumbosacral angle (Dub-LSA), which was used to evaluate lumbosacral kyphosis and defined as the angle between the L5 upper endplate and the posterior border line of S1 vertebrae. Dub-LSA < 90° was considered significant lumbosacral kyphosis [15].

**Fig. 2:**
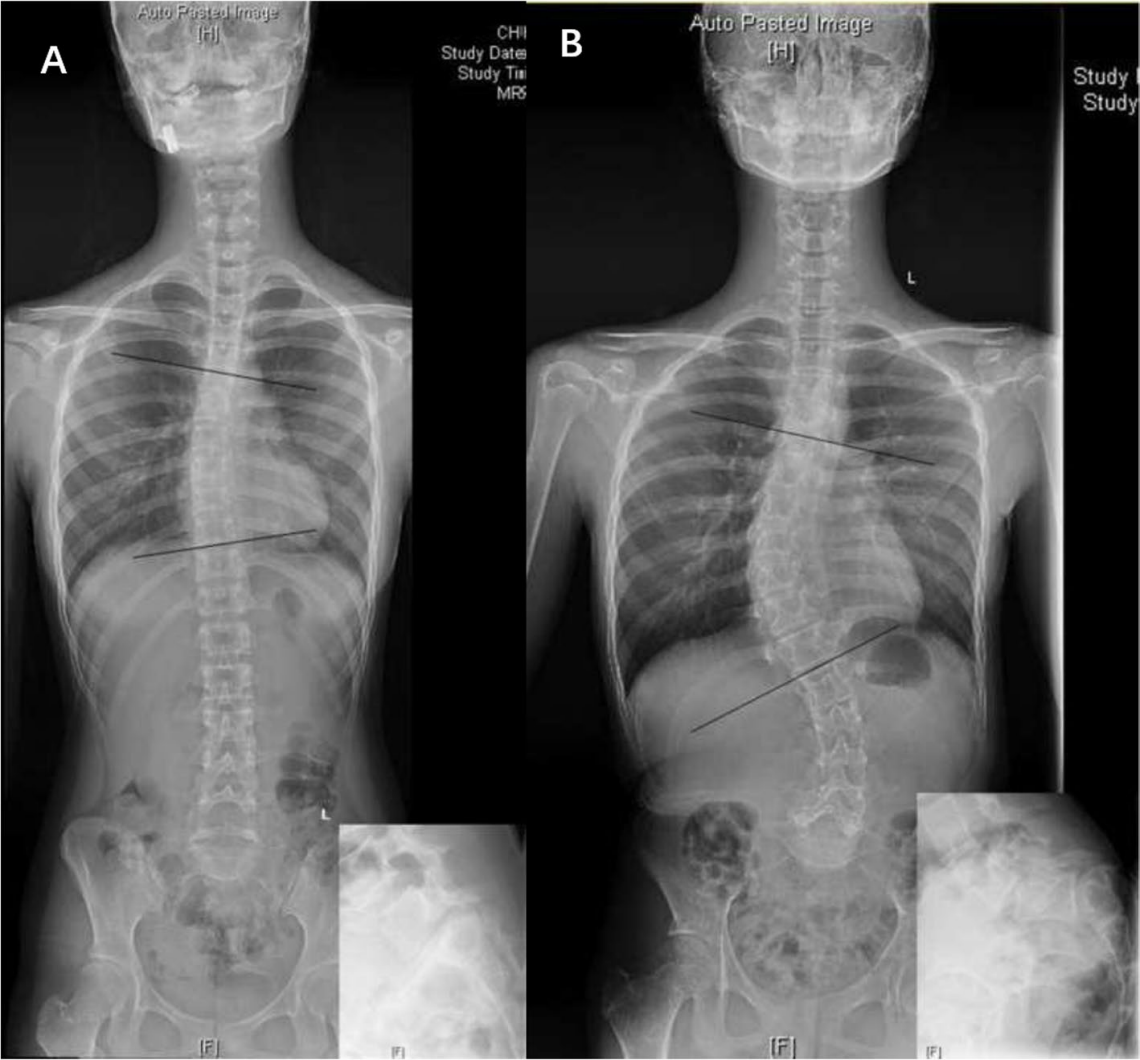
**A:** A 16-year-old female with dysplastic spondylolisthesis (Grade III) and adolescent idiopathic scoliosis (15°). **B:** A 12-year-old female with dysplastic spondylolisthesis (Grade V) and syndromic scoliosis (44°); she was diagnosed with Marfan Syndrome before surgery

**Fig. 3:**
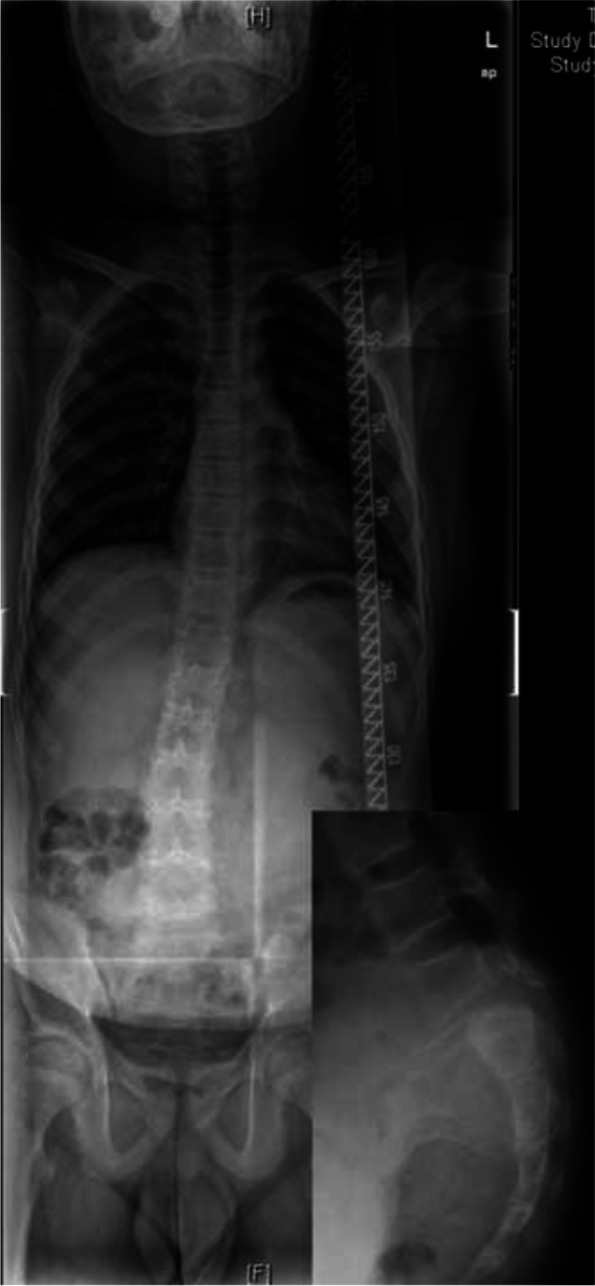
A 12-year-old male with dysplastic spondylolisthesis (Grade III) and spasm scoliosis with a tilted body, long curve span, and no vertebral rotation

**Fig. 4:**
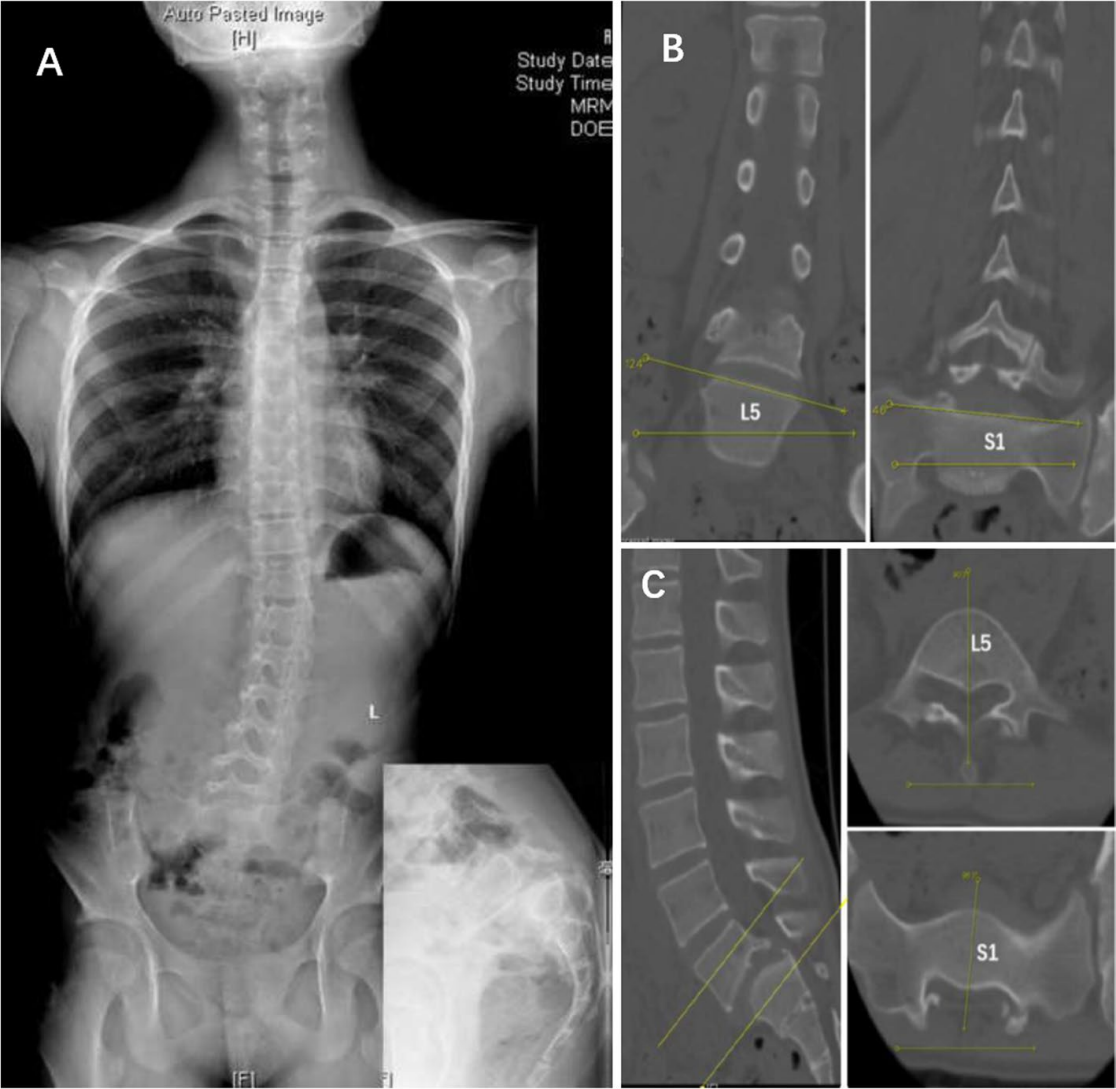
A 12-year-old female with dysplastic spondylolisthesis (Grade III) and olisthetic spondylolisthesis. **A:** Preoperative radiograph shows the lower lumbar curve with lower lumbar vertebral tilt and rotation. **B:** Coronal reconstructive CT scan showing L5 vertebra tilt relative to S1. **C:** Axial CT scan showing L5 rotation relative to S1

Patients with scoliosis were divided into three groups:(1) “independent” scoliosis (IS) group, in which scoliosis was independent from spondylolisthesis, such as adolescent idiopathic scoliosis (AIS), congenital scoliosis, syndromic scoliosis, etc. (Fig. 2); (2) spasm scoliosis (SS) group, in which scoliosis was typically with a tilted spine, long curve span, and low vertebral rotation value (Fig. 3); and (3) olisthetic scoliosis (OS) group, in which scoliosis was caused by asymmetric slippage with typically more rotation of the olisthetic vertebra (Fig. 4). In cases where SS and OS were difficult to distinguish on radiographs, computed tomography (CT) scans were used for determination of subtype. If CT showed L5 rotation (≥ 5°) with respect to S1 on the axial plane or tilt (≥ 5°) on the coronal plane, scoliosis was considered the OS type.

Radiographic parameters and patient-reported outcomes were collected before and after surgery and compared between groups. The visual analog scale (VAS), Oswestry Disability Index (ODI), and Japanese Orthopedic Association (JOA)-29 scores were used to evaluate clinical outcomes.

Independent sample t-tests were used to compare normally distributed data between groups. For non-normally distributed data, the Mann–Whitney rank sum test was adopted. Analysis of variance and Bonferroni’s test were used for comparison between multiple groups. Paired sample t-tests were used to compare the preoperative and postoperative data. The χ2 test was used to compare rates. SPSS version 21.0 (IBM Corporation, Armonk, NY, USA) was used for all statistical analyses. *P* < 0.05 was considered to be statistically significant.

## Results

Of the total patients, 56% (28/50) showed scoliosis. The general characteristics, spinal sagittal parameters, and QOL data of the 50 dysplastic spondylolisthesis patients are shown in Table 1. The slip percentage of patients with scoliosis was significantly higher than that of patients without scoliosis (70.8 ± 22.7% vs. 52.7 ± 19.8%, *P* = 0.005) and they also showed a smaller Dub-LSA angle than patients without scoliosis (61.8° ± 15.4° vs. 70.8° ± 11.2°, *P* = 0.025). The two groups did not differ significantly in age, PI, PT, SS, LL, preoperative VAS, ODI, or JOA scores (Table 2). Of the 28 patients with scoliosis, 8 were IS type (average Cobb angle 24.7° ± 15.2°), including 1 patient with Marfan syndrome, 1 patient with congenital scoliosis, and 6 patients with idiopathic scoliosis; 11 patients were SS type (average Cobb angle 13.9° ± 3.0°); and 9 patients were OS type (average Cobb angle 12.9° ± 1.9°). Coronal imbalance was observed in 3/8 (37.5%) patients in the IS group, 3/11 (27.3%) patients in the SS group, and 3/9 (33.3%) patients in the OS group. The comparison of parameters among the scoliosis subtype groups is shown in Table 3. The age of the OS group was significantly higher than SS group (Bonferroni’s test, *P* = 0.029), the preoperative Cobb angle of the IS group was significantly higher than SS group (Bonferroni’s test, *P* = 0.031) and OS group (Bonferroni’s test, *P* = 0.023), and the curve span of the SS group was significantly higher than that in the OS group (Bonferroni’s test, 8.0 ± 1.5 vs. 5.1 ± 0.9, *P* < 0.001).

**Table 1:** General data, spinal sagittal parameters, and QOL data of the 50 dysplastic spondylolisthesis patients

	**Pre-operation**	**Post-operation**	**P value**
Age (year)	14.9 ± 5.6		
Sex (male/female)	6/44		
Slip percentage (%)	63.6 ± 23.4	16.0 ± 18.3	< 0.001
Dub-LSA(°)	65.8 ± 14.3	85.2 ± 16.1	< 0.001
PI (°)	70.3 ± 11.9	72.8 ± 10.9	0.027
PT (°)	37.2 ± 8.9	31.3 ± 8.5	< 0.001
SS (°)	32.9 ± 12.9	41.5 ± 10.2	< 0.001
LL (°)	48.5 ± 19.2	47.3 ± 9.7	0.665
SVA (mm) (*n* = 35)	54.1 ± 36.1	31.9 ± 28.9	0.004
VAS (low back pain or lower limb pain)	4.7 ± 1.8	1.2 ± 1.1	< 0.001
ODI (%)	32.3 ± 15.5	5.5 ± 6.1	< 0.001
JOA-29 score	17.2 ± 4.7	26.4 ± 2.9	< 0.001

Data are presented as mean ± standard deviation. *QOL*, quality of life, *Dub-LSA* Dubousset’s lumbosacral angle, *PI* pelvic incidence, *PT* pelvic tilt, *LL* lumbar lordosis, *SVA* sagittal vertical axis, *VAS* visual analogue score, *ODI* Oswestry disability index, *JOA* Japanese orthopaedic association

**Table 2:** Comparison of parameters between the scoliosis and non-scoliosis groups

	**Scoliosis group (*n* = 28)**	**Non-scoliosis group (*n* = 22)**	**P value**
Age (year)	14.8 ± 5.5	15.0 ± 5.9	0.890
Slip percentage (%)	70.8 ± 22.7	52.7 ± 19.8	0.005
Dub-LSA(°)	61.8 ± 15.4	70.8 ± 11.2	0.025
PI (°)	69.9 ± 12.4	70.8 ± 11.4	0.797
PT (°)	38.2 ± 10.4	35.9 ± 6.7	0.367
Sacral slope (°)	30.6 ± 15.1	34.5 ± 9.5	0.438
LL (°)	51.2 ± 21.4	45.1 ± 15.7	0.270
SVA (mm)	52.2 ± 36.1 (*n* = 26)	44.4 ± 34.2(*n* = 16)	0.490
VAS (low back pain or lower limb pain)	4.4 ± 2.1	5.0 ± 1.5	0.210
ODI (%)	29.8 ± 16.9	35.4 ± 13.3	0.207
JOA-29 score	17.7 ± 5.3	16.5 ± 3.8	0.380

Data are presented as mean ± standard deviation. *Dub-LSA* Dubousset’s lumbosacral angle, *PI* pelvic incidence, *PT* pelvic tilt, *LL* lumbar lordosis, *SVA* sagittal vertical axis, *VAS* visual analogue score, *ODI* Oswestry disability index, *JOA* Japanese orthopaedic association

**Table 3:** Comparison of parameters among the different scoliosis groups

	**Independent scoliosis group (*n* = 8)**	**Spasm scoliosis group (*n* = 11)**	**Olisthetic scoliosis group (*n* = 9)**	**F value**	**P value**
Age (year)	13.1 ± 1.8	12.7 ± 2.9	18.9 ± 7.8	4.602	0.020
Slip Percentage (%)	75.6 ± 27.4	65.8 ± 21.5	72.6 ± 21.0	0.449	0.643
Dub-LSA(°)	56.8 ± 19.2	64.5 ± 10.3	62.9 ± 17.6	0.590	0.562
Coronal Cobb angle (°)	24.7 ± 15.2	13.9 ± 3.0	12.9 ± 1.9	5.188	0.013
Curve span	6.4 ± 0.7	8.0 ± 1.5	5.1 ± 0.9	13.106	< 0.001
C7PL-CSVL (mm)	23.3 ± 24.7	16.6 ± 15.1	18.9 ± 9.4	0.357	0.703
PI (°)	67.1 ± 14.4	72.3 ± 13.1	69.4 ± 10.4	0.392	0.680
PT (°)	39.8 ± 8.1	38.6 ± 13.3	36.5 ± 8.8	0.213	0.810
Sacral slope (°)	27.4 ± 19.0	33.7 ± 16.5	33.0 ± 9.1	0.437	0.651
LL (°)	46.4 ± 21.4	48.3 ± 25.1	58.9 ± 16.2	0.849	0.428
SVA (mm)	65.4 ± 32.2	58.2 ± 44.3	31.6 ± 19.0	2.163	0.138
VAS (low back pain or lower limb pain)	5.0 ± 2.7	4.5 ± 1.9	3.8 ± 1.6	0.759	0.479
ODI (%)	36.1 ± 14.7	31.9 ± 20.5	21.7 ± 11.3	1.761	0.193
JOA-29 score	17.0 ± 4.8	16.5 ± 5.9	19.7 ± 4.7	0.963	0.395

Data are presented as mean ± standard deviation. *Dub-LSA* Dubousset’s lumbosacral angle, *C7PL-CSVL* c7 plumb line to central sacral vertical line, *PI* pelvic incidence, *PT* pelvic tilt, *LL* lumbar lordosis, *SVA* sagittal vertical axis, *VAS* visual analogue score, *ODI* Oswestry disability index, *JOA* Japanese orthopaedic association

The average follow-up time of the 28 cases with scoliosis was 58.3 ± 41.3 months (range 3–168 months). The patient who was followed up for only 3 months had SS, the scoliosis was relieved at follow-up. By the last follow-up, no scoliosis resolution was observed in the IS group, whereas all SS patients were relieved of scoliosis (Table 4). Among the nine OS group patients, four (44.4%) had scoliosis resolution. Among the five patients without scoliosis resolution, two had mild scoliosis progression such that the Cobb angle by last follow-up had increased by 5°–10° compared to that before surgery. No coronal imbalance was observed by the last follow-up, except in one patient with idiopathic scoliosis.

**Table 4:** Comparison of pre-operative Cobb angle and Cobb angle at last follow-up in the scoliosis group

	**Pre-op Cobb angle (°)**	**Last follow-up Cobb angle(°)**	**P value**
Independent scoliosis group(*n* = 8)	24.6 ± 15.1	23.3 ± 13.9	0.562
Spasm scoliosis group(*n* = 11)	13.9 ± 3.0	2.3 ± 2.1	< 0.001
Olisthetic scoliosis group(*n* = 9)	12.9 ± 1.9	10.6 ± 5.7	0.198

Data are presented as mean ± standard deviation. *Pre-op* pre-operation; *Post-op* post-operation

## Discussion

**Fig. 5:**
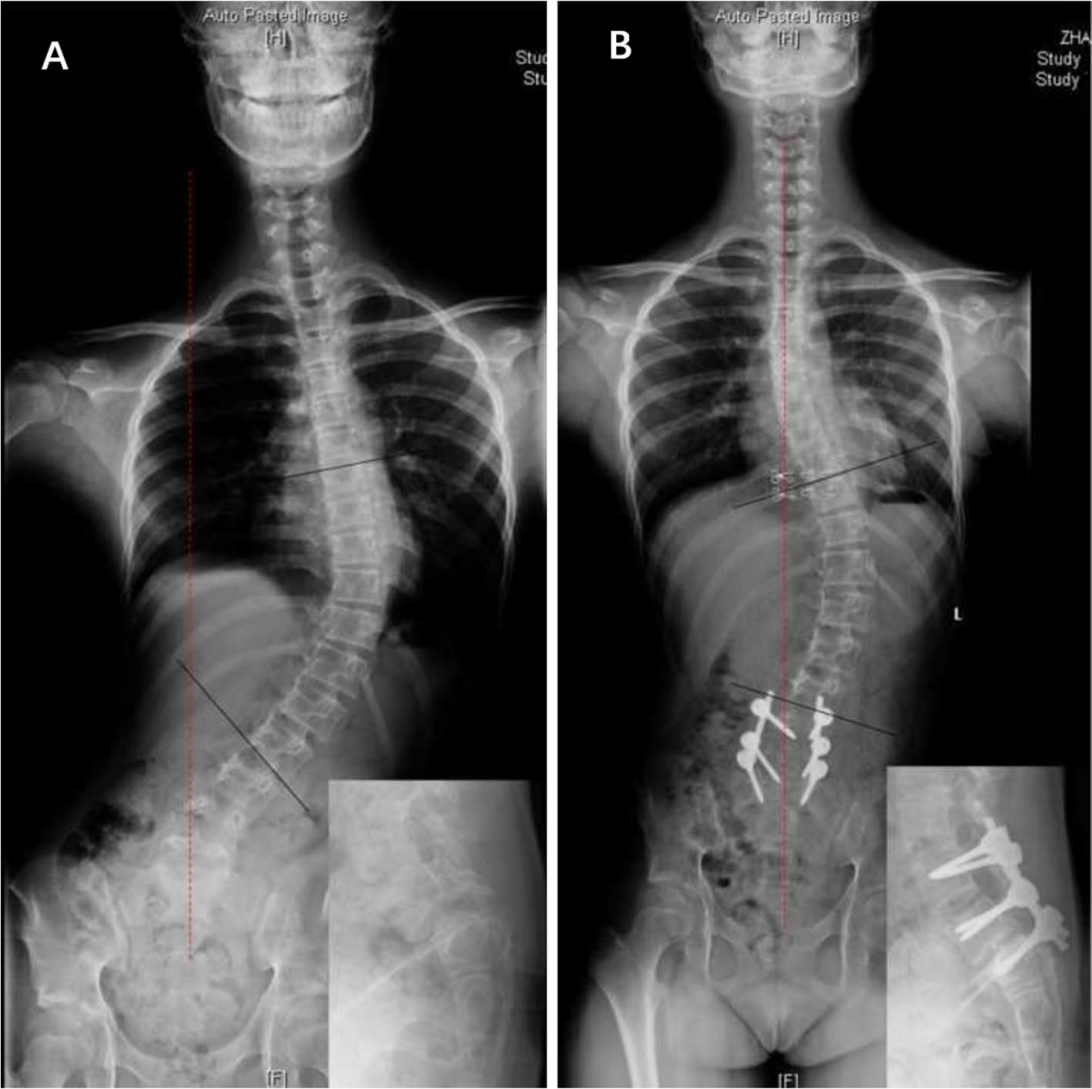
A 13-year-old female with dysplastic spondylolisthesis (grade III) and idiopathic scoliosis. **A:** Preoperative radiograph demonstrated obvious trunk tilt and vertebral rotation at the thoracolumbar spine; we believe the scoliosis was composed of idiopathic scoliosis and spondylolisthesis-induced scoliosis (54°). **B:** Postoperative radiograph at two-year follow-up shows relief of spondylolisthesis-induced scoliosis with normal coronal balance, while the idiopathic scoliosis was restored to its “original” shape (43°)

The results show that different types of scoliosis associated with spondylolisthesis may have different degrees of resolution after surgical treatment. Notably, 16% of the dysplastic spondylolisthesis patients had “independent” scoliosis (IS group) and the Cobb angle in this group was significantly larger than that of spondylolisthesis-induced scoliosis. Although the Cobb angle in the IS group was slightly reduced at the last follow-up compared with the preoperative angle, none of the patients in this group showed complete resolution (resolution rate 0%). Researchers agree that when spondylolisthesis is combined with idiopathic scoliosis, these two deformities should be treated separately [12]. Our findings are consistent with this conclusion. Furthermore, we found that some of these patients might have spondylolisthesis-induced scoliosis in addition to the pre-existing scoliosis, resulting in an increasing coronal Cobb angle and coronal imbalance (Fig. 5). For these patients, treatment of spondylolisthesis should be performed first to relieve the spondylolisthesis-induced scoliosis, and any remaining scoliosis should be observed for a period of time (we recommend 3–12 months) and treated according to the corresponding principles.

Spasm scoliosis is a functional curve caused by muscle contracture and often presents with a long curve span and no obvious rotation. Fixation and fusion may relieve pain and muscle spasm, thus relieving scoliosis. However, it is important to note that SS can take time to resolve after successful operation, sometimes a year or longer [12, 16].

In the present study, only 44% (4/9) of olisthetic curves were relieved after spondylolisthesis surgery. Du et al. [13] reported 56% resolution of OS and further demonstrated that failed olisthetic scoliosis resolution was related to an older age at surgery, a larger Cobb angle, and more severe L5 rotation. Interestingly, two patients with OS in our series experienced mild scoliosis progression during the follow-up period. This may be because we did not correct the rotation of the slipped vertebra and the “foundation” was fixed at an asymmetric position, thus leading to no relief of scoliosis or even progression of scoliosis with increasing age.

There may be scholars argue that SS is not actually a scoliosis but merely a lateral spinal deviation, but we think it is meaningful to define it as scoliosis. We can also call SS antalgic scoliosis as Crostelli et al. [12] called it. Indeed, SS is a functional scoliosis or functional curve. Thus, it is logic that SS will be dissolved if the causation is removed in time. If the causation persists long enough, the functional curve may become structural as there may be degenerative changes of the spine. This process may be similar to the very early stage of degenerative scoliosis. In most cases, SS and OS are both mild lumbar curves. However, it makes sense to distinguish between the two types because they may correspond to different treatment strategies. From our results, we can assume that decompression and solid fusion are enough to dissolve SS, and correction of L5 rotation and tilt should be considered for OS during the reduction of spondylolisthesis, as this may help to relive scoliosis and prevent curve progression.

According to the literature, most spondylolisthesis-induced scoliosis does not exceed 15°; however, due to the flexibility of the spine in adolescents and children, spondylolisthesis-induced scoliosis may sometimes be accompanied by a large Cobb angle (20° or more), which can be confused with idiopathic scoliosis [10, 12, 16–20]. It is important to distinguish the two cases, because the former can be relieved with reduction and fixation of the slip while the latter cannot. Several authors have reported complete resolution of severe scoliosis associated with spondylolisthesis after operative treatment of spondylolisthesis (Table 5) [17–20]. The scoliosis in these cases, with almost no vertebral rotation but large Cobb angle and marked coronal imbalance, should be considered as spasm scoliosis.

**Table 5:** Case reports that reported complete resolution of severe scoliosis associated with spondylolisthesis after operative treatment of spondylolisthesis

**Author**	**Pneumaticos et al. **[17]	**Zhou et al. **[18]	**Srivastava et al. **[19]	**Khashab et al. **[20]
Year of Publication	2003	2013	2016	2020
Age	17	12	12	12
Sex	Female	Female	Female	Female
Main complaints	Low back pain and right leg radiating pain	Progressively aggravating scoliosis without low back pain or leg pain	Low back pain, left L5 radiculopathy, and abnormal gait	Back pain, left side leg pain, spinal deformity and abnormal gait
Duration of symptoms (months)	6	24	NA	8
Degree of slippage	III	IV	IV	IV
Slip percentage	50%	88%	95%	> 75%
Pre-op Cobb angle(°) of scoliosis	30	50	44	28.8
Coronal imbalance (Yes/No)	Yes	No	Yes	Yes
Apex vertebra rotation (Nash-Moe method)	0	0	0	0
Operation	L4-S1 decompression, posterolateral fusion and instrumentation	L5-S1 decompression, reduction, circumferential fusion, and L4-S1 instrumentation	L5-S1 decompression, reduction, circumferential fusion, and L4-S2 instrumentation	decompression, partial reduction, circumferential fusion, and L4-S1 instrumentation
Follow-up time (months)	14	24	7	84
Scoliosis resolution (Yes/No)	Yes	Yes	Yes	Yes

Abbreviation: *Pre-op* pre-operation

In 2013, Crostelli et al. [12] summarized two main types of scoliosis associated with spondylolisthesis: (1) “idiopathic” scoliosis, in which scoliosis and spondylolisthesis are two different pathologies with no direct relation; and (2) spasm/antalgic scoliosis, which is caused by spondylolisthesis. They further classified spasm/antalgic scoliosis into two subtypes: pure spasm scoliosis, which is equivalent to spasm scoliosis described above, and spasm/olisthetic scoliosis, which is spasm scoliosis combined with olisthetic scoliosis, with more rotation in the olisthetic vertebra. The reason for this subsequent distinction is that, when we describe a lumbar curve presumably caused by spondylolisthesis, it is difficult to judge how much of the curve is due to muscular spasm and how much is due to the rotation “foundation” (the slipped vertebra) [12].

**Fig. 6:**
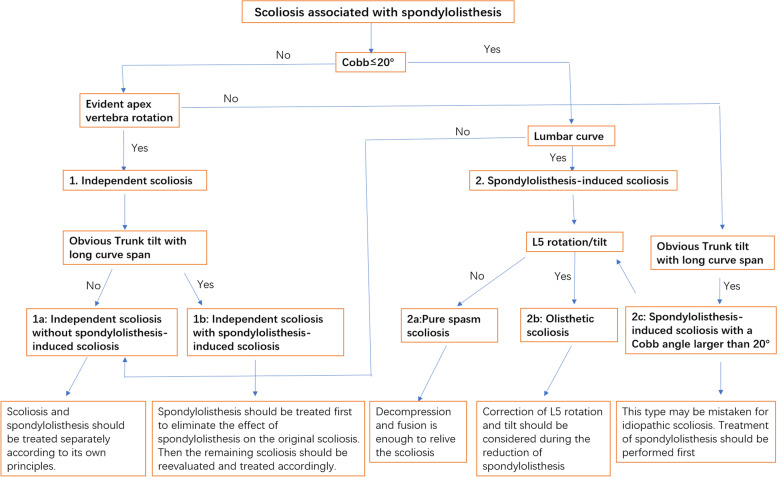
A flowchart to distinguish the different types of Modified Crostelli’s Classification of scoliosis associated with spondylolisthesis

**Fig. 7:**
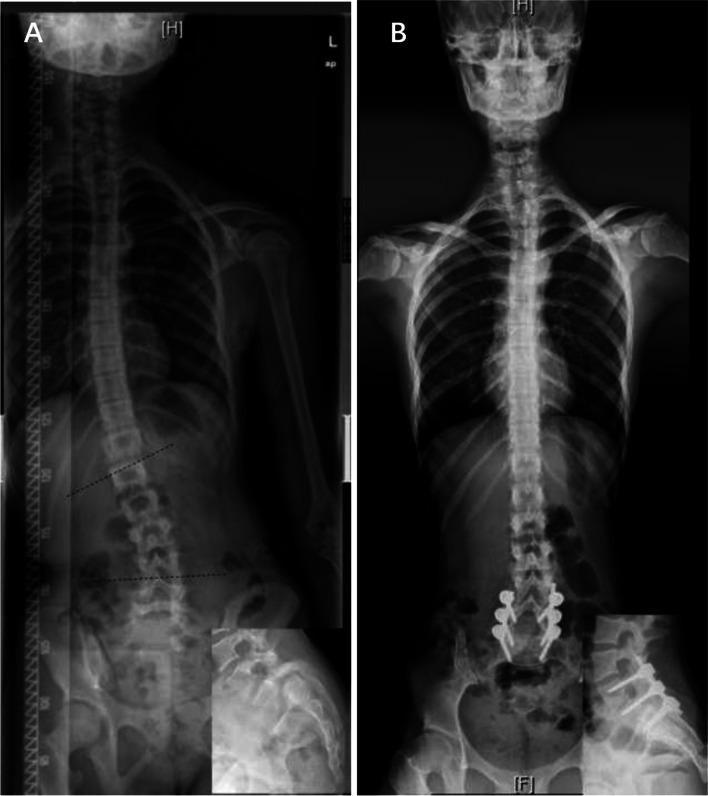
An 11-year-old female with dysplastic spondylolisthesis (grade IV) and sciatic scoliosis greater than 20°. **A:** The preoperative radiograph shows obvious scoliosis (24°) and coronal imbalance, without vertebral rotation. **B:** The one-year follow-up radiograph shows scoliosis resolution after spondylolisthesis surgery.

The relationship between spondylolisthesis and scoliosis is complex and sometimes it is not absolute. In our series of cases, we found that spondylolisthesis may be associated with different types of scoliosis at the same time and that it was sometimes difficult to distinguish between so-called “idiopathic” scoliosis and spondylolisthesis-induced scoliosis, especially when the Cobb angle was relatively large. Therefore, for scoliosis associated with dysplastic spondylolisthesis, we suggest the following modified Crostelli’s classification to better identify the type of scoliosis and guide treatment (Fig. 6):

1. “Independent” scoliosis, of which scoliosis and spondylolisthesis are two different pathologies, and scoliosis could be idiopathic, congenital, syndromic, etc.
  - “Independent” scoliosis without spondylolisthesis-induced scoliosis (Fig. 2): Typically, the curve is located in the thoracic or thoracolumbar region. Treatment of spondylolisthesis has no influence on scoliosis, and each disease should be treated separately according to its own principles.
  - “Independent” scoliosis accompanied by spondylolisthesis-induced scoliosis (Fig. 5): In most cases, the accompanying spondylolisthesis-induced scoliosis is pure spasm or so-called “sciatic”, resulting in aggravation of scoliosis. Thus, this type of scoliosis tends to have a large Cobb angle and obvious trunk tilt, with the maximum rotation at the apical vertebrae. Treatment of spondylolisthesis should be performed first to eliminate the effect of spondylolisthesis on the original scoliosis, and then the remaining scoliosis should be reevaluated and treated accordingly.
2. Spondylolisthesis-induced scoliosis, which is caused by the muscle spasm mechanism or/and the torsional “foundation” mechanism, usually has a lower curve value and is located in the lumbar region.
  - Pure spasm scoliosis, which is the most common type, with a Cobb angel no more than 20° and almost no vertebral rotation (Fig. 3). This type of scoliosis can be completely or largely relived after spondylolisthesis surgery, and the operative methods range from in situ fusion to complete reduction and fusion.
  - Olisthetic scoliosis, which is caused by L5 rotation and tilt during slipping, with or without spasm factor (Fig. 4). As the foundation of the spine, L5 rotation and tilt can cause a compensatory curve above L5, and the apex of the compensatory curve is usually located at lumbar region with mild vertebral rotation no more than Grade I (Nash-Moe Method). In this case, when performing a reduction procedure, attention should be paid to correct the rotation and tilt of L5, which may better relive the scoliosis.
  - Spondylolisthesis-induced scoliosis with a Cobb angle larger than 20°, which includes more muscular spasm factor, that is similar to pure spasm scoliosis (Fig. 7). In our opinion, this subtype distinction is necessary because, when we observe scoliosis with a Cobb angle > 20° associated with spondylolisthesis, it is not easy to determine whether the curve is independent or spondylolisthesis-induced. This type of scoliosis may be mistaken for idiopathic scoliosis. The key point to distinguish them is that idiopathic scoliosis has significant vertebral rotation at the apex that is matched with the Cobb angle, while spondylolisthesis-induced scoliosis has no or mild vertebral rotation at the apex and usually has significant coronal imbalance. The cases summarized in Table 5 should be classified as this subtype. Treatment of spondylolisthesis should be performed first. Successful decompression, reduction, and fusion often relieve this type of scoliosis.

This study is subject to several limitations. First, the sample size is small due to the rarity of dysplastic spondylolisthesis and the follow-up time for some cases is relatively short. Second, this study has the inherent limitations of a retrospective study and there may be recall bias. Third, there are no existing standard criteria to distinguish different types of scoliosis associated with spondylolisthesis. Thus, the distinction between different types of scoliosis in this study was based on previous studies and our own experience. Standard criteria for subtypes of scoliosis associated with spondylolisthesis still need to be developed.

## Conclusions

The incidence of scoliosis associated with dysplastic spondylolisthesis in young patients is approximately 60%, most of which is spondylolisthesis-induced scoliosis. Distinguishing different types of scoliosis may help surgeons plan treatment and understand prognosis. For spasm scoliosis, which is essentially a functional/nonstructural scoliosis, decompression and fusion of spondylolisthesis to relive the pain may fully correct the scoliosis. For Olisthetic scoliosis, correction of L5 rotation and tilt should be paid attention during reduction of spondylolisthesis. For dysplastic spondylolisthesis patients with associated significant scoliosis – whether “independent” or spondylolisthesis-induced – treatment of spondylolisthesis should be performed first and the remaining scoliosis should then be observed for a period of time and treated according to the corresponding principles.

## Data Availability

The datasets generated and/or analyzed during the current study are not publicly available due to the data containing information that could compromise patient privacy but are available from the corresponding author onreasonable request.

## Abbreviations

SS: Spasm scoliosis
OS: Olisthetic scoliosis
IS: Idiopathic scoliosis
QOL: Quality of life
CSVL: Central sacral vertical line
C7PL: C7 plumb line
PI: Pelvic incidence
PT: Pelvic tilt
SVA: Sagittal vertical axis
Dub-LSA: Dubousset’s lumbosacral angle
CT: Computed tomography
VAS: Visual analog score
ODI: Oswestry disability index
JOA: Japanese Orthopedic Association
